# Phenolic profile of different solvent extracts of *Reseda alba* L. and evaluation of anti-quorum sensing, antioxidant, and enzyme inhibition activities

**DOI:** 10.3389/fnut.2025.1699534

**Published:** 2025-11-28

**Authors:** Mohammed Nacer, Ali Kalla, Alfred Ngenge Tamfu, Sameh Boudiba, Karima Hanini, Soraya Hioun, Mohammed Messaoudi, Ayomide Victor Atoki, Selcuk Kucukaydin, Khattabi Latifa, Ozgur Ceylan, Louiza Boudiba

**Affiliations:** 1 Laboratory of Organic Materials and Heterochemistry (LOMH), Echahid Cheikh Larbi Tebessi University, Tebessa, Algeria; 2 Laboratory of Applied Chemistry and Renewable Energies (LACRE), Echahid Cheikh Larbi Tebessi University, Tebessa, Algeria; 3 Laboratory of Bioactive Molecules and Applications (LBMA), Echahid Cheikh Larbi Tebessi University, Tebessa, Algeria; 4 Department of Chemical Engineering, School of Chemical Engineering and Mineral Industries, University of Ngaoundere, Ngaoundere, Cameroon; 5 Food Quality Control and Analysis Program, Ula Ali Kocman Vocational School, Mugla Sitki Kocman University, Mugla, Türkiye; 6 Laboratoire de Recherche sur les Produits Bioactifs et Valorisation de la Biomasse, Département de Chimie, ENS Kouba, Alger, Algeria; 7 Department of Biochemistry, Kampala International University, Ishaka, Uganda; 8 Department of Medical Services and Techniques, Koycegiz Vocational School of Health Services, Mugla Sitki Kocman University, Mugla, Türkiye; 9 Biotechnology Research Center – CRBT, Constantine, Algeria

**Keywords:** medicinal legume, *Reseda alba*, phenolic profile, quorum-sensing inhibition, swarming inhibition, enzymes inhibition, antioxidant activity

## Abstract

**Background:**

*Reseda alba* (white mignonette) is a wild, edible, and medicinal plant native to the Mediterranean region, with limited studies on its chemical composition and bioactivities.

**Methods:**

The phenolic profile and bioassays of antioxidant, anti-swarming, quorum sensing (QS), and enzyme inhibitory activities of different solvent extracts of *R. alba* are investigated using high-performance liquid chromatography with diode-array detection (HPLC-DAD).

**Results:**

Rosmarinic acid was identified as the predominant phenolic compound in the ethyl acetate extract (197.5 ± 0.25 μg/g) and n-butanol extract (205.4 ± 0.47 μg/g). Minimal inhibitory concentrations (MICs) ranged from 0.3125 to 2.5 mg/mL against *Chromobacterium violaceum* and *Pseudomonas aeruginosa* PA01. These extracts demonstrated significant inhibition of quorum sensing and swarming motility against *C. violaceum* and *P. aeruginosa* PA01 at MIC and sub-MIC. Extracts exhibited inhibition of enzymes, especially cholinesterases implicated in neurodegenerative diseases. The extracts demonstrated antioxidant activity, as determined through six assays, with the dichloromethane extract (DCME) exhibiting higher antioxidant activity compared to the standards *α*-tocopherol and ascorbic acid in the ferric-reducing antioxidant power (FRAP) assay.

**Conclusion:**

The results demonstrate that *R. alba* extracts (RAEs) possess significant inhibitory effects on enzymes implicated in neurodegenerative disorders, such as Alzheimer’s disease, particularly through butyrylcholinesterase inhibition. Additionally, the extracts show promising anti-quorum sensing and anti-swarming activities, which could reduce microbial virulence and biofilm formation, suggesting potential as alternative antimicrobial agents. The moderate antioxidant activity further supports its therapeutic potential. Overall, *R. alba* could be developed as a natural source for managing enzyme-related diseases and controlling bacterial infections by targeting microbial communication mechanisms.

## 1 Introduction

*Reseda alba* (white mignonette) is a wild edible leguminous plant, and its young leaves are used as a vegetable and in salads in parts of Greece. The young inflorescence shoots of this plant are also edible. The tops of the shoots are eaten raw, seasoned with olive oil, or after being cooked and then stir-fried with garlic and olive oil. Parts of *R. alba* L. are medicinal and are typically considered to have blood-cleansing properties, which are beneficial for the liver. Medicinal plants are prominent sources of multifaceted drug development, as they contain a diverse array of chemical compounds that can target multiple illnesses. Although *R. alba* is traditionally valued for its culinary and medicinal uses in the Mediterranean region, especially for its blood-purifying and liver-supportive properties, detailed phytochemical profiling and evaluation of its biological activities remain limited. This gap highlights the need for a systematic investigation to better understand and validate its therapeutic potential. *R. alba* belongs to the Resedaceae family together with other important species such as *Reseda luteola* and *Reseda villosa* (80).

Enzyme inhibitors are one of the most interesting aspects of natural drug search, since any perturbation of their biochemical activities leads to immediate and defined pathogenic effects (1). The majority of the medications and diagnostic methods involve enzyme targets, and, therefore, enzyme inhibitors are crucial in medicine (2). Cholinesterases are enzymes that break down the neurotransmitter acetylcholine, creating a deficit implicated in Alzheimer’s disease (AD) according to the cholinergic hypothesis (3). Butyrylcholinesterase (BChE) and acetylcholinesterase (AChE) hydrolyze butyrylcholine and acetylcholine neurotransmitters, respectively, reducing nerve transmission to the brain and causing neurological disorders (4, 5). Alzheimer’s disease (AD) is a severe neurodegenerative disorder characterized by progressive brain deterioration, memory loss, and dementia, affecting over 25 million people worldwide (6). Although drugs such as donepezil, galantamine, and rivastigmine function as cholinesterase inhibitors, their limitations have shifted interest toward natural inhibitors of AChE and BChE (7, 8). Elevated plasma levels of AChE and BChE in patients with AD and type 2 diabetes contribute to brain imbalance and deregulation (5). Carbohydrates digested by *α*-amylase and α-glucosidase increase postprandial glucose levels in diabetic patients (9, 10). Inhibiting α-amylase slows the breakdown of carbohydrates into monosaccharides and offers a way to prevent diabetes. Tyrosinase, a copper-containing enzyme, triggers melanin production, which, when excessive, causes skin hyperpigmentation, browning of fruits and vegetables, and cancer risks (11). Tyrosinase inhibitors are therefore important in cosmetic skin whitening, medicinal skin treatments, and food preservation as anti-browning agents (12, 13). Oxidative stress, which leads to neuro-damage and senile plaque formation, plays a role in many diseases, including AD, suggesting antioxidants may help alleviate dementia (14). Natural antioxidants are incorporated into diets to combat oxidative stress, which can cause cell damage and promote disease progression (15). Additionally, antioxidants improve vascular function and insulin secretion, reduce insulin resistance, and regulate oxidative stress markers, thus helping to control glucose metabolism and improve diabetic status (16).

Virulence factors promote microbial resistance, pathogenicity, and severity of infections (17). Microbial virulence involves the ability to infect a host and cause diseases using molecules that enable cells to colonize the host, communicate, evade harsh conditions, host the immune system, and the presence of antibiotics (18). These virulence factors, including quorum sensing, which is a microbial cell-to-cell communication system that mediates many processes and biofilm formation, enables bacteria to develop a protective sheath composed of complex polysaccharides and proteins (17, 19).

Since ancient times, people have used plants as a source of medicine, providing humanity with a rich repertoire of bioactive compounds (20). These natural therapies have played an important role in treating or preventing a variety of disorders (21), providing a holistic approach to healthcare. From traditional herbal remedies (22) to modern pharmaceuticals derived from botanical sources (23), plant medicine, commonly referred to as herbal medicine or phytotherapy (24), continues to intrigue researchers, healthcare professionals, and individuals seeking alternative and complementary therapies (25). Various plant species have had their extracts tested for bioactive chemicals such as tannins, flavonoids, triterpenoids, phytosterols, peptides, polysaccharides, saponins, alkaloids, and more (26, 27). The hitherto undiscovered *R. alba* plant stands out in this category as a noteworthy source of bioactive substances that have not been studied in terms of their phytochemical content or potential biological effects (28, 29). Multiple studies have demonstrated that extracts from various plants in the same *Resedaceae* family have strong biological action (30, 31). *R. alba* (leaves, stems, roots, and seeds) may possibly have potential biological activities, especially antioxidant, antibacterial, and anti-enzyme potential, which requires scientific evaluation.

The objective of this work was to identify the major phenolic compounds in extracts of *R. alba* and evaluate their inhibitory effects on cholinesterases, tyrosinases, and *α*-amylase, as well as their antioxidant, anti-quorum sensing, and antibiofilm properties.

## 2 Materials and methods

### 2.1 Plant material and extraction

The plant material was harvested in April 2018 during its flowering period in the El Ghourira Mountains, situated at 35°18′00.1″N and 8°05′53.2″E in Tebessa, Algeria. Soraya Hioun of Echahid Cheikh Larbi University’s Department of Natural and Life Sciences (living things and CJB/African Plant Database) identified specimen plants of *R. alba* L., which belongs to the Resedaceae family, using taxonomic references (32, 33). Voucher samples were deposited under the number RES/2.1.2/HS in the herbarium collection of the Laboratory of Applied Chemistry and Renewable Energies (LACRE) of the same university. One kilogram of the plant cotyledons (leaves, stems, and roots) was cut into small pieces and immersed in an aqueous alcohol solution (methanol/water, 7/3, v/v) for 24 h. Tthe obtained mixture was then filtered and evaporated until dryness. After this step, to remove the chlorophyll and fats, boiled water was added to the resulting crude extract and kept at room temperature for 12 h. After that, a liquid–liquid extraction process was realized using several solvents, starting with dichloromethane, ethyl acetate, and n-butanol, followed by a gradient increasing polarity. The resulting organic phases for each solvent were evaporated to dryness using a rotary evaporator (34, 35) to afford dichloromethane extract (DCME, 0.087%), ethyl acetate (EAE, 0.169%), and n-butanol [butanol extract (BE), 0.145%], which were stored and used for further studies.

### 2.2 High-performance liquid chromatography with diode-array detection (HPLC-DAD) determination of phenolic compounds

The method was slightly modified to analyze the phenolic compounds (36). The methanol and water extracts were dissolved in methanol and water, filtered using an Inertsil ODS-3 reverse-phase C_18_ column (5 μm, 250 mm, 4.6 mm i.d.), and separated at a temperature of 40 °C using the same Inertsil ODS-3 reverse-phase C_18_ column. The sample injection volume was 20 μL, and the solvent flow rate was 1.0 mL/min. Tow mobile phases A and B were prepared using 0.5% acetic acid each, and created the gradients in the following manner: 0–10% B (up to 0.01 min), 10–20% B (0.01–5 min), 20–30% B (5–15 min), 30–50% B (15–25 min), 50–65% B (up to 25 min), 65–75% B (up to 40 min), 75–90% B (up to 50 min), and 90–10% B (up to 55 min) (37). A photodiode array detector was used, with a preferred wavelength 280 nm, and the retention times of each phenolic compound and ultraviolet (UV) data were utilized for identification. There were three parallel analyses. For the quantitative analysis of phenolic compounds, calibration curves were generated by injecting standard compounds at known concentrations (0.0, 0.00782, 0.01563, 0.03125, 0.0625, 0.125, 0.25, 0.5, and 1.0 ppm). A total of twenty-six standard phenolic compounds were used. Ascending in order, they were p-hydroxybenzoic, chlorogenic, 3-hydroxybenzoic, *trans*-cinnamic, p-coumaric, protocatechuic, syringic, gallic, ellagic, rosmarinic, ferulic acids, kaempferol, hesperetin, pyrocatechol, vanillin, 6,7-dihydroxycoumarin, coumarin, myricetin, rutin, luteolin, apigenin, catechin, quercetin, chrysin, hesperetin, and taxifolin. All identified and quantified compounds were expressed in micrograms per gram (μg/g) extract dry weight.

### 2.3 Microbial strains used and minimum inhibitory concentration (MIC) determination

In this investigation, three different microorganisms were used: *Chromobacterium violaceum* CV12472, *Pseudomonas aeruginosa* PA01, and *C. violaceum* CV026. The recommended microtiter broth dilution procedure was used to calculate the minimum inhibitory concentration (MIC) (38). Mueller-Hinton Broth (MHB) was used as the assay medium with a bacterial density of 5 × 10^5^ colony-forming units (CFU)/mL. Different extract concentrations (2, 1, 0.5, 0.25, 0.125, 0.0625, and 0.03125 mg/mL) were combined with 100 μL of cell suspension and inoculated into the microplate wells. Following inoculation, the microplates were incubated at 37 °C for 24 h before being examined. Each experiment was performed in triplicate, and the MIC was established as the lowest amount of extract that was unable to produce observable proliferation.

### 2.4 Violacein inhibition method using *Reseda alba* CV12472

The capacity of all extracts to inhibit quorum sensing (QS) in *C. violaceum* ATCC 12,472 was tested qualitatively (39, 40). Sterile microplates containing 200 μL of Luria–Bertani (LB) broth were used for the experiment. To these plates, 10 μL of an overnight culture of *C. violaceum* adjusted to 0.4 at 600 nm was added. The plates were then incubated for 24 h at 30 °C in both the presence and absence of sub-minimum inhibitory concentrations (MIC) of extracts. The formation of the violacein pigment was found to be decreasing, and the absorption at 585 nm was assessed. Experiments were repeated in triplicate to ensure the reproducibility of the results, and the percentage of violacein inhibition was estimated using the following Equation 1 (41):


$$\text{Violacein inhibition}\;(\%)=\{\frac{\begin{array}{l} \mathrm{OD}\;585\;\text{control}\\ -\mathrm{OD}\;585\;\text{sample} \end{array}}{\mathrm{OD}\;585\;\text{control}}\}\times 100\;$$ (1)

### 2.5 *Pseudomonas aeruginosa* PA01 swarming motility inhibition assay

As previously mentioned, the swarming motility inhibition test (42) was carried out. *P. aeruginosa* PAO1 strain overnight culture was injected at the center of swarming plates containing 1% peptone, 0.5% agar, 0.5% NaCl, and 0.5% filter-sterilized D-glucose, with a range of sample extract concentrations (50, 75, and 100 g/mL in an aqueous solution). The plates were then incubated for 18 h at an appropriate temperature and in an upright position. The control experiment was carried out identically, but without extracts. By following the fronts of swarming bacterial cells, the swarming migration could be observed.

### 2.6 Bioassay evaluation of the quorum-sensing inhibition (QSI) using *Chromobacterium violaceum* CV026

The evaluation of the QSI was conducted following the procedure outlined in the literature (43). For that, 5 mL of Soft Top Agar (STA, 2.0 g of tryptone with 1.3 g of agar, and 1.0 g of sodium chloride in 200 mL of deionized water). After that, 100 μL of the CV026 overnight culture was seeded into the STA, and then 20 μL of Acyl Homoserine Lactone (AHL) was added. After carefully stirring, a thin layer of the resultant mixture was directly applied to the surface of a solid LBA (Luria Bertani Agar) plate. Following the solidification of the overlay, wells with a diameter of 5 mm were drilled into each plate, and each well contained 50 μL of filtered and sterilized extract. 45 μL of sterile LB broth and 5 μL (100 μg/mL) of C10 HSL (N-decanoyl-L-homoserine lactone) were added to the positive control well. The extracts were diluted from 1:1 to 1:16 using LB broth to establish the activity detection limit. Each experiment was repeated three times, and the endpoint was a minimal dilution of the extract that inhibited the synthesis of violacein. A pronounced halo around the well showed the presence of antimicrobial activity, whereas a cream-colored or white halo indicated the occurrence of QSI. Subsequently, the test plates were incubated at 30 °C for 3 days.

### 2.7 Anticholinesterase activity assessment

To determine the anticholinesterase activity via the enzymatic inhibition of acetylcholinesterase and butyrylcholinesterase, spectrophotometry was utilized in the previously described method (44, 45). A mixture of 130 μL of sodium phosphate buffer (100 mM, pH 8.0), 10 μL of sample extract solution (dissolved in ethanol at various concentrations), and 20 μL of buffer enzyme solution (either AChE or BChE) was incubated at 25 °C for 15 min. Afterward, 20 μL of 0.5 mM 5,50-dithiol-bis (2-nitrobenzoic) acid (DTNB) was added, followed by 20 μL of 0.71 mM acetylthiocholine iodide or 20 μL of 0.2 mM butyrylthiocholine chloride to initiate the reaction. The formation of a yellow 5-thio-2-nitrobenzoate anion was then monitored at 412 nm. This was done to measure the enzymatic hydrolysis of either butyrylthiocholine chloride or acetylthiocholine iodide and the subsequent release of thiocholine in the presence of DTNB. The percentage of enzyme inhibition was then calculated for an extract concentration of 200 μg/mL.

### 2.8 Anti-tyrosinase activity assay

To evaluate the anti-tyrosinase activity of RAEs, a fungal tyrosinase enzyme was used. First, 20 μL of the enzyme (150 μL, 100 mM, pH 6.8) was added to 10 μL of different concentrations of each extract solution (dissolved in ethanol), then incubated for 10 min at room temperature. After that, 20 μL of L-DOPA was added to this mixture as a substrate, and the change in absorbance at 475 nm was monitored as an indication of the enzymatic reaction caused by chromium DOPA formation. The percentage of tyrosinase reaction inhibition was calculated using the same equation used for AChE/BChE inhibition activity. Kojic acid was used as a reference standard inhibitor for comparison (46, 47).

### 2.9 *α*-Amylase inhibitory assay

The α-amylase inhibitory activity of the investigated extracts was accomplished according to a standard technique described in previous studies (45, 48). 50 μL of ethanolic extract solution was added to 150 μL of a prepared mixture containing: 1.5 mg of soluble starch with buffered solution (0.2 M, pH 6.8), and 150 μL of NaCl (17 mM), followed by the addition of 10 μL of alpha-amylase (25 units/mL). The resulting mixture was incubated at 37 °C for 30 mi. Subsequently, 20 μL of NaOH (2 N) and 20 μL of the color reagent (3, 5-dinitrosalicylic acid (DNS, 44 μmoL)), 106 μmoL of potassium sodium tartrate tetrahydrate, and 40 μmoL of NaOH were added. After that, a second incubation for 20 min at 100 °C was performed. The absorbance of the resulting mixture was measured at 540 nm using the Parkin Elmer Multi-Reader.

### 2.10 Antioxidant activity

This study employed six distinct methods to ascertain the antioxidant content in the extracts. These assays were performed in 96-well microdishes using a PerkinElmer. multimode microplate reader. Concentrations providing 50% inhibition are half maximal inhibitory concentration (IC_50_) and A0.50, and methanol served as a negative control, and both values were graphically compared to the efficiency of the extracts with the standards. The experiments were realized in triplicate, and the values were recorded as the average of these values.

#### 2.10.1 ABTS^•+^ scavenging assay

The experiment used a spectrophotometric approach that has already been reported in the literature, but with some changes (49, 50) Two stock solutions were prepared, one containing potassium persulfate (2.4 mM) and the other containing 2,20-azino-bis(3-ethylbenzothiazoline-6-sulfonic acid) (ABTS). Both solutions were mixed and allowed to remain at room temperature in the dark for 12–16 h before being used so as to generate the radical cation (ABTS^•+^). A combination of 160 μL of ABTS^•+^ and 40 μL of the test solution (extracts) or standards—which were butylated hydroxylanisole (BHA) and butylated hydroxyltoluene (BHT) in methanol—was incubated for 10 min. The absorbance was measured at 734 nm, and the percentage inhibition was calculated using the following Equation 2:


$$\%\text{ABTSscavenging}=[(A_{\text{control}}-A_{\text{sample}})∕A_{\text{control}}]\times 100\;$$ (2)

where $A_{\text{sample}}\;$ is the absorbance of the ABTS radical cation in the sample solution and $A_{\text{control}}\;$ is its absorbance in methanol.

#### 2.10.2 DPPH*^•^* radical scavenging activity

This experiment aims to see whether the investigated extract could reduce the violet-colored 2,2-diphenyl-1-picrylhydrazyl radical (DPPH^•^) radical (51, 52). Varying concentrations of extract samples were tested. A solution of 40 μL of extract of standard (BHA and BHT) in methanol and 160 μL of the DPPH^•^ solution previously prepared by dissolving 0.06 mg in 1.0 mL of methanol was mixed and incubated in the dark for 30 min. The absorbance was measured at 517 nm. The results were expressed as IC_50_ values.

#### 2.10.3 Galvinoxyl free radicals (GOR) scavenging assay

Using the galvinoxyl radical scavenging procedure, the potential of *R. alba* extracts (RAEs) to inhibit activity was evaluated (53). A 40 μL volume from each dilution of the sample solution (plant extracts or standard) was added to 160 μL of galvinoxyl (0.1 mmoL in methanol). After that, the combination was kept in the dark at room temperature for 120 min. At 428 nm, the absorbance values were measured and the findings were expressed as IC_50_ values, which were then compared with those of BHA and BHT.

#### 2.10.4 Cupric reducing antioxidant capacity (CUPRAC)

The CUPRAC test was used to assess the extract’s ability to decrease copper ions (54). For that, 50 μL of 10 mM CuCl_2_, 50 μL of 7.5 mM Neocuproine (C_14_H_12_N_2_), and 60 μL of ammonium acetate (NH_4_CH_3_CO_2_, pH = 7.0 buffer, 1 M) were added to each well of a 96-well microplate containing 40 μL of the sample. The microplate was checked for absorbance at 450 nm after 1 h of incubation. The results were expressed as absorbances, and the concentration giving an absorbance of 0.5 (A_0.5_) expressed in μg/mL was determined from the absorbance curves at different concentrations. BHA and BHT served as the reference antioxidants for comparison.

#### 2.10.5 Phenanthroline reduction assay

The phenanthroline procedure described in the literature (55) was used to evaluate the antioxidant potential of the investigated extracts. To sum up, 50 μL of FeCl_3_ (0.2%) and 10 μL of various dilutions of different sample solutions (extract or standard in methanol) were mixed. Then, 5 μL of O-phenanthroline (0.5%) was added, and the volume was adjusted with 110 μL of methanol. The mixture was then incubated for 20 min at 30 °C, and the absorbance was measured at 510 nm. The outcomes were expressed as an A_0.50_ value and compared with the antioxidant-positive controls (BHA and BHT).

#### 2.10.6 Ferric-reducing antioxidant power assay

The tests realized for the evaluation of the ferric-reducing antioxidant power (FRAP) exerted by *R. alba* extracts were elaborated following the protocol described in the literature (56). 50 μL of potassium ferricyanide (1%) was added to 40 μL of phosphate buffer (pH = 6.6; 0.2 M) and 10 μL of the sample dissolved in methyl alcohol (extract or standard). At 50 °C, the mixture was incubated for 20 min. After the addition of 50 μL of trichloroacetic acid solution (10%), 40 μL of distilled water, and 10 μL of ferric chloride solution (0.1%). The absorbance was then measured at 700 nm, and the results were presented as A_0.5_ values, which were contrasted against those of ascorbic acid and *α*-tocopherol.

### 2.11 Statistical analysis

Activity assays were performed in triplicate. The data were recorded as means ± Standard Error of the Means (SEM). Minitab 16 statistical software was used to determine the significant differences between means using one-way analysis of variance (ANOVA), in which *p* < 0.05 was regarded as significant.

## 3 Results

### 3.1 HPLC-DAD phenolic composition

**Figure 1:**
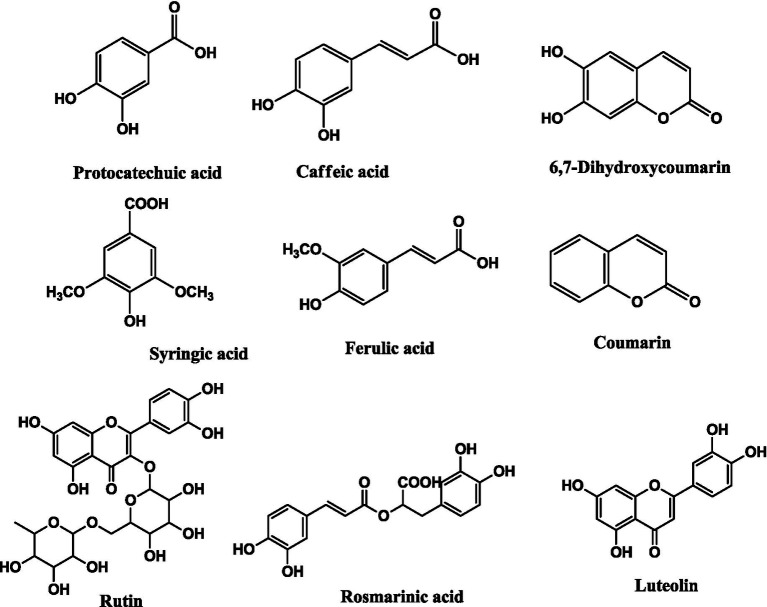
Structures of phenolic compounds identified in *R. alba* extracts.

The HPLC-DAD method was employed to identify and quantify the phenolic compounds in the plant extracts, and the results are presented in Table 1. Nine phenolic compounds were identified and quantified in the extracts. Protocatechuic acid (0.72 ± 0.02 μg/g), 6.7-dihydroxy coumarin (1.52 ± 0.05 μg/g), caffeic acid (11.74 ± 0.17 μg/g), syringic acid (1.71 ± 0.08 μg/g), ferulic acid (6.60 ± 0.27 μg/g), rutin (0.45 ± 0.02 μg/g), and luteolin (2.10 ± 0.11 μg/g) were identified in the DCME extract. Caffeic acid, coumarin, rutin, and luteolin were detected in the ethyl acetate and butanol extracts in almost the same amounts. The only difference between these two extracts was in the amounts of rosmarinic acid, which were 197.5 ± 0.25 μg/g and 205.4 ± 0.47 μg/g in the ethyl acetate extract (EAE) and butanol extract (BE), respectively. The structures of the identified phenolic compounds in *R. alba* extracts are given in Figure 1.

**Table 1:** Phenolic composition of the extracts by HPLC-DAD (μg/g)^a^.

No	Phenolic compounds	RT (min)	DCME	EAE	BE
1	Gallic acid	5.70	–	–	–
2	Protocatechuic acid	8.75	0.72 ± 0.02	–	–
3	Catechin	10.68	–	–	
4	Pyrocatechol	11.04	–	–	
5	Chlorogenic acid	12.35	–	–	
6	*p*-Hydroxybenzoic acid	12.77	–	–	
7	6,7-Dihydroxycoumarin	14.10	1.52 ± 0.05	–	
8	Caffeic acid	15.09	11.74 ± 0.17	6.09 ± 0.15	6.38 ± 0.22
9	3- Hydroxybenzoic acid	15.98	–	–	–
10	Syringic acid	16.56	1.71 ± 0.08	–	–
11	Vanillin	17.78	–	–	–
12	*p*-Coumaric acid	20.56	–	–	–
13	Taxifolin	21.26	–	–	–
14	Ferulic acid	22.14	6.60 ± 0.27	–	–
15	Coumarin	24.49	–	18.54 ± 0.36	20.63 ± 0.42
16	Rutin	25.30	0.45 ± 0.02	11.48 ± 0.30	11.73 ± 0.51
17	Ellagic acid	26.11	–	–	–
18	Rosmarinic acid	26.77	–	197.5 ± 0.25	205.4 ± 0.47
19	Myricetin	27.35	–	–	–
20	Quercetin	30.83	–	–	–
21	*trans*-Cinnamic acid	31.33	–	–	–
22	Luteolin	31.70	2.10 ± 0.11	5.19 ± 0.16	5.25 ± 0.19
23	Hesperetin	32.14	–	–	
24	Kaempferol	33.21	–	–	
25	Apigenin	33.77	–	–	
26	Chrysin	38.40	–	–	–

a Values expressed are means ± SEM of three parallel measurements (*p* < 0.05). –, not detected.

### 3.2 Inhibition of swarming motility, violacein, and quorum sensing by *R. alba* extracts

Before testing for anti-QS properties, the MIC values of the extracts were determined and reported in Table 2. MIC values varied from 0.625 (EAE) to 2.5 (DCME) against *C. violaceum* CV12472, and EAE had the best violacein inhibition of 100 ± 0.0 (MIC) and 16.8 ± 0.4 (MIC/8). Against *C. violaceum* CV026, MIC values were 0.3125 for EAE and BE and 0.625 for DCME. All extracts inhibited QS against *C. violaceum* CV026 almost to the same extent at MIC and MIC/2, as shown in Table 2. MIC values against *P. aeruginosa* PA01 were 1.25 (DCME) and 0.625 (EAE and BE). The DCME showed the best anti-swarming effects, with concentration-dependent percentage inhibitions varying from 55.9 ± 1.3 (MIC) to 10.5 ± 0.8 (MIC/4) as shown in Table 2.

**Table 2:** Inhibition of violacein, quorum sensing, and swarming motility.

Test bacteria	Concentration	DCME	EAE	BE
*C. violaceum* CV12472	MIC (mg/mL)	2.5	0.625	1.25
		Violacein inhibition (%)		
	MIC	60.5 ± 1.1	100 ± 0.0	65.8 ± 1.3
	MIC/2	30.9 ± 1.0	66.1 ± 0.8	33.7 ± 0.4
	MIC/4	09.5 ± 0.2	35.7 ± 0.5	15.3 ± 0.5
	MIC/8	–	16.8 ± 0.4	–
*C. violaceum* CV026	MIC (mg/mL)	0.625	0.3125	0.3125
		Quorum-sensing inhibition zone diameters (mm)		
	MIC	11.0 ± 0.3	12.5 ± 0.2	11.5 ± 0.5
	MIC/2	07.5 ± 0.1	9.0 ± 0.7	8.5 ± 0.6
	MIC/4	–	–	–
	MIC/8	–	–	–
*P. aeruginosa* PA01	MIC (mg/mL)	1.25	0.625	0.625
		Swarming inhibition (%)		
	MIC	55.9 ± 1.3	39.0 ± 1.1	34.5 ± 0.9
	MIC/2	28.5 ± 0.7	17.9 ± 0.5	15.3 ± 0.1
	MIC/4	10.5 ± 0.8	8.4 ± 0.2	–
	MIC/8	–	–	–

### 3.3 Enzyme inhibitory activities of *Reseda alba* L. extracts

The results of the extracts’ inhibition of tyrosinase, *α*-amylase, AChE, and BChE are shown in Table 3. It is important to note that the maximum test concentration was 200 μg/mL, and compounds with IC_50_ values higher than this did not display up to 50% inhibition within this range. Galantamine presented an IC_50_ value of 5.50 ± 0.18 μg/mL in the AChE assay and an IC_50_ of 42.38 ± 0.50 μg/mL in the BChE test. As shown in Table 3, the three extracts had IC_50_ AChE inhibition values of 51.52 ± 1.37 μg/mL for DCME, 61.02 ± 0.98 μg/mL for EAE, and 48.2 ± 0.28 μg/mL for BE. BChE inhibition IC_50_ values were 43.01 ± 0.28 μg/mL, 13.68 ± 0.84 μg/mL, and 31.43 ± 8.26 μg/mL for the DCME, EAE, and BE, respectively. For the enzyme tyrosinase inhibition, the IC_50_ values of the standard acid Kojic were 23.80 ± 0.25 μg/mL, and those for the investigated extracts were 62.28 ± 1.76 μg/mL for DCME, 78.83 ± 1.91 μg/mL for EAE, and 61.08 ± 1.02 μg/mL for BE. The best activity for *α*-amylase inhibition was observed at 25.08 ± 0.03 μg/mL for DCME compared to the standard acarbose, which had an IC_50_ value of 36.93 ± 10.70 μg/mL.

**Table 3:** Enzyme inhibitory activity potential of *Reseda alba* L. extracts: inhibition (%) at 200 μg/mL and IC_50_ (μg /mL).

Test sample	AChE		BChE		Tyrosinase		α-Amylase	
	Inhibition (%)	IC_50_ (μg/mL)	Inhibition (%)	IC_50_ (μg/mL)	Inhibition (%)	IC_50_ (μg/mL)	Inhibition (%)	IC_50_ (μg/mL)
DCME	114.45 ± 1.02	51.52 ± 1.37	51.96 ± 3.54	43.01 ± 0.28	11.19 ± 0.16	62.28 ± 1.76	95.78 ± 4.35	25.08 ± 0.03
EAE	91.12 ± 1.04	61.02 ± 0.98	73.57 ± 0.74	13.68 ± 0.84	32.21 ± 0.75	78.83 ± 1.91	28.13 ± 5.36	>200
BE	102.1 ± 0.95	48.2 ± 0.28	58.07 ± 2.71	31.43 ± 8.26	31.88 ± 0.85	61.08 ± 1.02	25.26 ± 5.10	>200
Galantamine	83.43 ± 0.67	5.50 ± 0.18	76.51 ± 0.31	42.38 ± 0.50	NT	NT	NT	NT
Kojic acid	NT	NT	NT	NT	75.27 ± 0.56	23.80 ± 0.25	NT	NT
Acarbose	NT	NT	NT	NT	NT	NT	37.21 ± 3.54	36.93 ± 10.70

Values represent the means ± SEM of three simultaneous sample measurements (*p* < 0.05). NT, not tested. Inh. (%): percentage inhibition.

### 3.4 Antioxidant activity of *R. alba* L. extracts

In this study, the ferric-reducing antioxidant power (FRAP), phenanthroline assays, cupric reducing antioxidant capacity (CUPRAC), 2,2-diphenyl-1-picrylhydrazyl radical (DPPH^•^), 2,2′-azino-bis(3-ethylbenzothiazoline-6-sulfonic acid) radical cation (ABTS^•+^), and galvinoxyl radical (GOR) were carried out, with the results shown in Table 4.

**Table 4:** Antioxidant activity of *Reseda alba* extracts.

Test sample	FRAP assay	Phenanthroline assay	CUPRAC assay	DPPH^•^ assay	ABTS^•+^ assay	GOR assay
	A_0.50_ (μg/mL)	A_0.50_ (μg/mL)	A_0.50_ (μg/mL)	IC_50_ (μg/mL)	IC_50_ (μg/mL)	IC_50_ (μg/mL)
DCME	4.30 ± 0.43	61.11 ± 0.91	78.22 ± 1.80	26.16 ± 1.54	31.33 ± 0.96	50.12 ± 0.12
EAE	37.77 ± 0.58	48.82 ± 1.68	86.59 ± 1.62	88.66 ± 2.07	13.57 ± 1.14	47.76 ± 1.24
BE	94.33 ± 2.57	49.98 ± 1.26	69.70 ± 1.00	72.14 ± 1.44	22.74 ± 0.81	38.47 ± 0.86
BHA	NT	0.93 ± 0.07	3.64 ± 0.19	6.14 ± 0.41	1.03 ± 0.00	5.38 ± 0.06
BHT	NT	2.24 ± 0.17	9.62 ± 0.87	12.99 ± 0.41	1.59 ± 0.03	3.32 ± 0.18
α-Tocopherol	34.93 ± 2.38	NT	NT	NT	NT	NT
Ascorbic acid	6.77 ± 1.15	NT	NT	NT	NT	NT

Values expressed are means ± SEM of three simultaneous measurements (*p* < 0.05). NT, not tested.

The results of the FRAP, phenanthroline, and CUPRAC assays were reported as A_0.50_, which corresponds to the concentration at 0.5000 absorbances. The amount of substance required to achieve 50% inhibition in the DPPH^•^, ABTS^•+^, and GOR assays determines the IC_50_ or A_0.50_ values. The DCME was the most active extract in the ferric-reducing antioxidant power (FRAP) assay, with the lowest A_0.50_ value of 4.30 ± 0.43 μg/mL, compared to *α*-tocopherol and Ascorbic acid, which had A_0.50_ values of 34.93 ± 2.38 and 6.77 ± 1.15 μg/mL, respectively. At the same time, the EAE activity was close to standard α-tocopherol with an A_0.50_ value of 37.77 ± 0.58 μg/mL. In the phenanthroline assay, the EAE extract exhibited the highest activity among the extracts, with the lowest A_0.50_ of 48.82 ± 1.68 μg/mL compared to BHA (0.93 ± 0.07 μg/mL) and BHT (2.24 ± 0.17 μg/mL). In the CUPRAC assay, the BE was most active, with an A_0.50_ value of 69.70 ± 1.00 μg/mL. The standards BHA and BHT had A_0.50_ values of 3.64 ± 0.19 and 9.62 ± 0.87 μg/mL, respectively. In the DPPH^•^ radical scavenging assay, DCME was the most active extract with the lowest IC_50_ value of 26.16 ± 1.54 μg/mL as compared to BHA (5.73 ± 0.41 μg/mL) and BHT (12.99 ± 0.40 μg/mL). In the ABTS^•+^ assay, the EAE showed the best activity with an IC_50_ value of 13.57 ± 1.14 μg/mL, which is relatively close to that of the standards BHA (IC_50_ = 1.03 ± 0.00 μg/mL) and BHT (IC_50_ = 1.59 ± 0.03 μg/mL). In the GOR assay, the BE was the most active, exhibiting an IC_50_ value of 38.47 ± 0.86 μg/mL, while the standards BHA and BHT had IC_50_ values of 5.38 ± 0.06 and 3.32 ± 0.18 μg/mL, respectively. DCME was the most active extract in the FRAP and DPPH^•^ assays, while EAE was the most active in the phenanthroline and ABTS^•+^ assays. The BE exhibited higher activity compared to the other extracts in the CUPRAC and GOR assays.

## 4 Discussion

Plants produce a variety of structural phenolic compounds, including phenolic acids, lignans, stilbenes, and different flavonoids. These chemicals have important roles in diet and health, including shielding tissues from oxidative stress (57). Numerous studies have been conducted on the biological activities, extractive and analytical properties, and protective effects of polyphenols against UV light, pests, and other stressors. Additionally, phenolic compounds provide appealing scents and pigments (58, 59). Owing to the significance of phenolic compounds in food and health, the phenolic composition of *R. alba* extracts was prepared by maceration and liquid–liquid extraction, and their phenolic profiles were examined by means of HPLC-DAD. This is an appropriate technique for polyphenol extraction, and it is typically impacted by solvent polarity (60). The polar solvents ethyl acetate and butanol extracted similar phenolic compounds, as shown in Table 1. The method of detecting and measuring phenolic compounds can be suitably performed using HPLC-DAD, as in this study. Depending on the planting region, environment, and harvest season, the plant *R. alba* typically contains phenolic compounds in varying amounts. Phenolic compounds and their glycosilonates have been described in reseda species and particularly in *R. alba* (61, 62). In the present study, protocatechuic acid, 6,7-dihydroxycoumarin, caffeic acid, syringic acid, ferulic acid, coumarin, rutin, rosmarinic acid, and luteolin were identified in *R. alba* from Algeria, the polar extracts (EAE and BE) were found to be abundantly rich in rosmarinic acid. Rosmarinic acid is well-documented for its potent antioxidant, anti-inflammatory, antimicrobial, and neuroprotective properties, which likely contribute significantly to the observed antioxidant and enzyme inhibitory activities. Specifically, rosmarinic acid has been shown to scavenge free radicals, inhibit cholinesterase enzymes, and modulate signaling pathways associated with neurodegenerative diseases (81).

Pathogenic bacteria utilize quorum sensing to generate virulence factors, such as violacein production and swarming motility, both of which can be examined at sub-MIC concentrations to ensure selective pressure on the bacterial cells (63). The effects of the extracts on the inhibition of violacein and quorum sensing could be attributed to the phenolic compounds and other constituents they contain. The EAE was particularly effective in showing the highest violacein inhibition against *C. violaceum* CV12472 compared to the other extracts. Although the phenolic compounds identified in the EAE and BE were the same, their activities differed, suggesting that some unidentified phytoconstituents may contribute to their bioactivities. Violacein is used as a signal molecule for communication through QS in bacteria. According to previous studies, quorum sensing (QS) regulates virulence factors in bacteria, including motility, violacein synthesis, and biofilm formation (12). The majority of these virulence factors also play a role in the development of microbial resistance mechanisms. Therefore, the search for quorum-quenching extracts and chemicals can help to reduce the emergence of microbial resistance and treat diseases caused by drug-resistant pathogens (64). It is therefore noteworthy to highlight that *R. alba* extracts at MIC and sub-MIC concentrations were able to inhibit some virulence factors, such as violacein production and swarming motility. The bacteria *C. violaceum* CV12472 produces violacein when growing normally, and inhibiting violacein production in this strain implies inhibition of signal molecule production. However, the mutant strain *C. violaceum* CV026 does not produce violacein unless an external hormone source is supplied to it. In the QSI assay, the *R. alba* extracts prevented these bacteria from producing violacein even when the acylhormoserine lactone (AHL) was used as an external hormone source. This model implies signal reception disruption. Inhibiting violacein production in both models suggests that the extracts prevent both signal molecule production and signal molecule reception (65). Bacteria employ various motilities, such as swarming, to locate nutrient sources, evade stress and antibiotics, and also move and colonize surfaces prior to forming biofilm (66). The inhibition of swarming motility by *R. alba* extracts indicates their potential in reducing the spread of bacteria on surfaces and preventing the establishment of biofilms. Flagellated bacteria, such as *P. aeruginosa* PA01, are suitable for this assay.

AChE, BChE, tyrosinase, and *α*-amylase enzymes were tested for the first time for the inhibition ability of *R. alba* extracts: DCME, EAE, and BE. These important enzymes have been linked to a wide range of human diseases (67), including diabetes for α-amylase, Parkinson’s disease for tyrosinase (68), and Alzheimer’s disease for AChE and BChE (69, 70). Galantamine, kojic acid, and acarbose were used as positive controls for cholinesterases, tyrosinases, and α-amylase, respectively. The results in Table 1 showed that the studied extracts have important activity. In the BChE inhibitory activity assay, the EAE and BE performed best on the galantamine standard, respectively. In the AChE inhibitory activity assay, the EAE had nearly the same activity as the galantamine standard. The high activity of EAE could be attributed to the high amounts of rosmarinic acid. Rosmarinic acid (RA) has been shown to suppress amyloid *β* (Aβ) plaques in the brains of mice induced with AD, revealing enhancement of the dopamine-signaling pathway and increased levels of monoamines (82). The neuroprotective potential of rosmarinic acid is typically achieved by modulating pro-inflammatory cytokine expression and preventing neurodegeneration, as well as reducing damage, with a possible nanotechnological approach in treating neurodegeneration (83).

The DCME outperformed the acarbose standard in the *α*-amylase inhibitory activity assay. In the tyrosinase inhibitory activity assay, the BE worked the best when compared to the standard, which was kojic acid. Some of the phenolic chemicals in these extracts are bioactive and have been shown to work against cholinesterase and stop other enzymes from working (60). According to studies, extracts that inhibit α-amylase can help manage diabetes by lowering blood glucose levels after eating meals. Inhibiting the α-amylase enzyme that hydrolyzes carbohydrates into sugars is an effective method of lowering blood glucose levels and diabetic symptoms (71). Recently, the neuroprotective effects of natural compounds with anti-inflammatory and antioxidant activities have been clearly demonstrated, especially using phenolic compounds such as rosmarinic acid (83).

Antioxidants are substances that have the ability to neutralize free radicals and reactive oxygen species, particularly in the human body, and their actions lower the risk of many diseases (72). Medicinal plant extracts can be evaluated in various ways to assess their effectiveness as antioxidants (73). Natural antioxidants present in plants can inhibit or prevent the formation of reactive oxygen species (74, 75), which, in turn, protect the human body from oxidative stress. In this study, we evaluated the antioxidant activities of DCME, EAE, and BE from the *R. alba* plant using six different complementary methods: FRAP assay, phenanthroline assay, CUPRAC assay, DPPH assay, ABTS radical scavenging assay, and galvinoxyl radical (GOR) assay. In the six assays, the decrease in absorbance of free radicals was attributed to the natural antioxidants present in the extract, which can scavenge radicals through hydrogen donation (76). According to our findings, which are presented in Table 2, the results were close to those of several standard antioxidants used in the assay, making them significant (*p* < 0.05). The DCME outperformed the ascorbic acid standard in the FRAP assay. The EAE was the most active sample in the phenanthroline and ABTS^+•^ assays. Accordingly, in the CUPRAC and GOR assays, the BE performed the best when compared to the standard, which was BHA and BHT. In terms of the DPPH^•^ assay, the DCME had nearly the same activity as the BHT standard. The phenolic compounds can be linked to the anti-free radical actions of ABTS^+•^, DPPH^•^, and galvinoxyl, as well as the CUPRAC, phenanthroline, and FRAP assays. From these findings, it appears that the phenolic compounds in *R. alba* extracts may act as antioxidants, free radical scavengers, hydrogen sources, oxygen singlet extinguishers, and metal ion chelators. Protection against certain diseases is the most significant function of antioxidants, such as phenolic compounds, which protect cells against damage caused by free radicals by combining with them to form stable, neutral, and harmless compounds (77). Due to their antioxidant activity, naturally occurring polyphenols are believed to reduce the risk of several serious illnesses, including cancer and cardiovascular disease. For this reason, research on antioxidant compounds from foods and other natural sources of medicine has become increasingly important (78). Since natural medicinal plant antioxidants are thought to be safer than synthetic ones, it is necessary to use and produce more potent versions of these antioxidants (79, 84). The complex chemical composition of the plant in this study includes phenolic chemicals present in it at various levels and capable of cooperating to induce a variety of behaviors.

This study presents promising *in vitro* bioactivities of *R. alba* extracts; however, several limitations must be acknowledged. The lack of *in vivo* validation means that the pharmacokinetics, safety, and efficacy in whole organisms remain unknown. Additionally, the use of crude extracts limits precise identification of the active compounds responsible for the observed effects. Mechanistic insights into the molecular pathways underlying quorum-sensing inhibition, enzyme inhibition, and antioxidant activities were not explored in this work. Future research should focus on isolating and characterizing pure bioactive compounds from *R. alba*, conducting comprehensive *in vivo* studies to assess therapeutic potential and safety, and exploring the development of this plant as a nutraceutical or natural therapeutic agent. These steps will help to better understand and harness the medicinal potential of *R. alba*.

## 5 Conclusion

A new species, *R. alba* L., was named and characterized in this study. This species is used in traditional Algerian medicine as a diuretic and digestive cure. The results suggest that this species has a high concentration of rosmarinic acid. *R. alba’s* antioxidant, antibacterial, and enzyme-inhibiting properties indicate great potential of this plant. The extracts inhibited microbial virulence factors such as violacein production, QS, and swarming motility at low concentrations (MIC and sub-MIC). The extracts inhibited AChE, BChE, tyrosinase, and *α*-amylase, which are enzymes whose overexpression can lead to diseases. Six complementary assays, including FRAP, Phenanthroline, CUPRAC, DPPH^•^, ABTS^•+^, and GOR, were used to evaluate antioxidant activity, which ranged from low to moderate. Further research, including the isolation of pure compounds from this plant, will be necessary to identify the compounds responsible for the observed bioactivities.

## Data Availability

The original contributions presented in the study are included in the article/supplementary material, further inquiries can be directed to the corresponding author.
